# The Monocyte to Macrophage Transition in the Murine Sterile Wound

**DOI:** 10.1371/journal.pone.0086660

**Published:** 2014-01-22

**Authors:** Meredith J. Crane, Jean M. Daley, Olivier van Houtte, Samielle K. Brancato, William L. Henry, Jorge E. Albina

**Affiliations:** Department of Surgery, Rhode Island Hospital and The Warren Alpert School of Medicine of Brown University, Providence, Rhode Island, United States of America; Duke University Medical Center, United States of America

## Abstract

The origin of wound repair macrophages is incompletely defined and was examined here in sterile wounds using the subcutaneous polyvinyl alcohol sponge implantation model in mice. Phenotypic analysis identified F4/80^+^Ly6C^hi^CD64^+^MerTK^–^ monocytes and F4/80^+^Ly6C^low^CD64^+^MerTK^+^ macrophages in the wound. Circulating monocytes were the precursors of inflammatory Ly6C^hi^ wound monocytes. Ly6C^low^MerTK^+^ macrophages appeared later, expressed CD206, CD11c, and MHC class II, produced cytokines consistent with repair function, and lacked a gene expression profile compatible with mesenchymal transition or fibroblastic transdifferentiation. Data also demonstrated that Ly6C^hi^ wound cells were precursors of Ly6C^low^ macrophages, although monocytes did not undergo rapid maturation but rather persisted in the wound as Ly6C^hi^MerTK^–^ cells. MerTK-deficient mice were examined to determine whether MerTK-dependent signals from apoptotic cells regulated the maturation of wound macrophages. MerTK-deficient mice had day 14 cell compositions that resembled more immature wounds, with a smaller proportion of F4/80^+^ cells and higher frequencies of Ly6G^+^ neutrophils and Ly6C^hi^ monocytes. The cytokine profile and number of apoptotic cells in day 14 wounds of MerTK-deficient mice was unaffected despite the alterations in cell composition. Overall, these studies identified a differentiation pathway in response to sterile inflammation in which monocytes recruited from the circulation acquire proinflammatory function, persist in the wound, and mature into repair macrophages.

## Introduction

Tissue injury induces an inflammatory response that results in the recruitment of polymorphonuclear leukocytes and monocytes to the site of damage. Circulating monocytes are recruited to wounded tissues as macrophage precursors [1]–[9]. The experimental depletion of wound monocyte/macrophage populations has revealed their essentiality to the repair process [10]–[13]. The order and timing by which infiltrating blood monocytes acquire macrophage traits in the sterile wound remains incompletely defined. This monocyte-to-macrophage transition was investigated using cells isolated from subcutaneously implanted polyvinyl alcohol (PVA) sponges in mice.

A recent publication challenged the traditional view that monocytes which extravasate into tissues obligatorily differentiate into macrophages or dendritic cells (DCs) [14]. In that report, specific populations of blood monocytes were shown to migrate into normal skin, lungs, and lymph nodes, where they retained monocyte markers without acquiring the molecular signature of macrophages or DCs. Data to be presented indicate that monocytes migrating into a site of sterile inflammation, here an experimental wound, can either persist as monocytes with a pro-inflammatory phenotype or differentiate *in situ* to macrophages capable of producing mediators associated with repair.

To examine the transition from monocyte to macrophage in the wound, F4/80^+^ cells were monitored for the acquisition of a CD64^+^Mer tyrosine kinase (MerTK)^+^ macrophage signature phenotype, as recently defined by the Immunological Genome Project [15]. Co-expression of CD64 and MerTK on F4/80^+^ cells allows for the distinction of macrophages from CD64^+^MerTK^low/neg^ monocytes [14]. Results demonstrated that monocytes arriving in the early wound remained MerTK^low/neg^ until day 3 after wounding, and rapidly transitioned to early pro-inflammatory monocytes through acquisition of CD14 expression and TNF-α production. MerTK expression on F4/80^+^ wound cells increased over time and was accompanied by loss of Ly6C expression. Ly6C^low^MerTK^+^ wound macrophages were capable of releasing pro-repair mediators, including VEGF and TGF-β, and evidence to be presented suggests that this population arose from the maturation of Ly6C^hi^ pro-inflammatory monocyte/macrophage precursors.

Reports by others have demonstrated the upregulation of fibroblast and myofibroblast markers, including procollagens and α-smooth muscle actin, in inflammatory myeloid cells, suggesting that wound macrophages may undergo transdifferentiation during the repair process [16]–[18]. This potential fate of wound macrophages was assessed by examining genes associated with fibroblastic and/or mesenchymal transition.

MerTK is a member of the Tyro3/Axl/Mer (TAM) receptor tyrosine kinase family, whose functions include the phagocytosis and clearance of apoptotic cells by macrophages [19]–[24], and the subsequent dampening of inflammatory responses [25]–[29]. Previous work from this laboratory revealed that wound macrophages can induce apoptosis in neutrophils, which are recruited in large numbers to early wounds, and ingest the apoptotic debris [30]–[32]. Based on the known functions of MerTK, its role in mediating the transition from an inflammatory to a reparative wound monocyte/macrophage phenotype was also examined.

The work described here demonstrates that MerTK^+^ wound macrophages arise from the maturation of inflammatory monocytes recruited from the circulation. Monocytes entered the wound rapidly after injury, where they persisted before acquiring a repair macrophage phenotype. MerTK deficiency slightly affected the monocyte-to-macrophage transition by altering the cell composition, although not the cytokine environment, of wounds. Overall, these studies identified two functionally and phenotypically distinct myeloid cell subsets in the wound, and demonstrate that these populations are related along a maturation pathway.

## Materials and Methods

### Ethics Statement

All animal studies were carried out according to the Guide for the Care and Use of Animals of the National Institutes of Health and were approved by the Rhode Island Hospital Institutional Animal Care and Use Committee (Protocol number 0146-12). Surgical procedures were performed under isofluorane anesthesia and all necessary steps to minimize suffering were taken.

### Mice

All mice were housed in pathogen-free facilities at Rhode Island Hospital. C57BL/6J (B6; CD45.2), B6. SJL-Ptprc^a^Pepc^b^/BoyJ (CD45.1), B6;129-Mertk^tm1Gr1^/J (MerTK^−/−^), and B6129SF2/J (MerTK control) mice were obtained from The Jackson Laboratory (Bar Harbor, ME). Heterozygous CX_3_CR1-gfp/+ (CX_3_CR1-GFP) mice were generated by breeding homozygous male B6.129P-*Cx3cr1^tm1Litt^*/J mice (CX_3_CR1-gfp/gfp; The Jackson Laboratory) to female C57BL/6J mice (The Jackson Laboratory). Male mice were used for experiments at 8–10 weeks of age.

### Polyvinyl Alcohol Sponge Insertion and Wound Cell Isolation

All surgery in mice was performed under anesthesia by isofluorane inhalation. Backs were shaved and cleaned with iodopovidone solution and 70% EtOH. Six 1 cm×1 cm×0.6 cm polyvinyl sponges (Ivalon) were placed subcutaneously through a midline dorsal incision under sterile conditions, and the incision closed with surgical clips. At indicated time points, mice were euthanized by CO_2_ asphyxiation prior to sponge removal. Cells were isolated from sponges using a Stomacher (Tekmar) in HBSS medium (1% FBS/Penicillin-Streptomycin/Hepes), washed, and red blood cells lysed. Cell yield and viability was determined by trypan blue exclusion in a hemocytometer. Sterility of the model has been confirmed by routine culture of sponges following removal from the mouse.

### Blood Cell Isolation

Mice were euthanized by CO_2_ asphyxiation and blood was obtained by cardiac puncture using heparinized syringes. Leukocyte-containing buffy coats were isolated following centrifugation of whole blood in Wintrobe tubes. Red blood cells were lysed, and blood leukocyte yield and viability was determined by trypan blue exclusion using a hemocytometer.

### Identification of Monocytes/Macrophages by Flow Cytometry

Markers used to identify monocyte/macrophage subsets included: F4/80-PerCP Cy5.5 or APC (clone BM8; eBioscience & Biolegend), Ly6C-FITC, PE or APC-Cy7 (clone AL-21; BD Biosciences), CCR2-PE or APC (Clone 475301; R&D Systems), MHC class II-PE (clone M5/114.15.2; BD Biosciences), CD11c-APC (clone HL3; BD Biosciences), CD206-PE (C-type Mannose Receptor 1, clone MR5D3; AbD Serotec), CD14-PE (clone Sa2–8; eBioscience), MerTK-APC (clone 108928; R&D Systems) and CD64-PE (clone X54-5/7.1.1; BD Biosciences). Surface FcγRII/III was blocked with 2.4G2 antibody (BD Biosciences). Cells were surface-stained for 30 minutes on ice in staining buffer (1x DPBS/1% FCS). Where indicated, cells were fixed for 30 minutes, treated with 1x permeabilization wash (Cytofix/Cytoperm Kit; BD Biosciences) and intracellularly stained with TNF-α-PE (clone MP6-XT22; BD Biosciences).

### Intracellular Staining of *in vivo* Phagocytosed Neutrophils

To measure phagocytosis of neutrophils by flow cytometry, wound cells were stained with Ly6C-FITC and F4/80-APC to identify macrophages and with Ly6G-V450 (clone 1A8; BD Bioscience) to exclude neutrophils. Cells were fixed and permeabilized as described above, then intracellularly stained with Ly6G-PE (clone 1A8; BD Biosciences). Doublets were excluded from analysis and isotype controls for surface and intracellular Ly6G antibodies were used to ensure specificity of intracellular Ly6G staining.

### Flow Cytometric Analysis of Caspase-3/7 Activity

Measurement of activated caspase-3/7 was performed using the Vybrant® FAM Caspase-3 and -7 Assay Kit for flow cytometry according to manufacturers instructions (Invitrogen).

Samples were acquired on a FACSCalibur using CellQuest software or on a FACSAria using FACSDiva software (BD Biosciences). Data were analyzed with FlowJo software (Tree Star Inc.). Analysis gates were determined using isotype controls or appropriate negative controls where indicated.

### Cell Sorting and Macrophage Culture

Macrophages were enriched from isolated wound cells by negative selection. Specifically, cells were stained with Ly6G-PE (clone 1A8; BD Biosciences), CD2-PE (clone RM2–5; BD Biosciences), TER119/Erythroid Cells-PE (clone Ter-119; BD Biosciences), and/or Siglec-F (MerTK^−/−^ experiments; clone E50–2440; BD Biosciences) for ≤1 hour, washed, and incubated with anti-PE magnetic beads (Miltenyi Biotec) for 20 minutes. Cells were washed and eluted through a magnetic column (Miltenyi Biotec) to deplete non-monocyte/macrophage populations. Enriched monocytes/macrophages were assessed for viability by trypan blue exclusion and surface stained with Ly6C-FITC and F4/80-APC as described above. Eosinophils were identified as F4/80^int^SSC^hi^ and were excluded during sorting. Ly6C^hi^F4/80^+^ and Ly6C^low^F4/80^hi^ populations were sorted to greater than 90% purity under sterile conditions on a FACSAria (BD Biosciences). Sorted cells were assessed for viability by trypan blue exclusion, resuspended in complete medium (DMEM/5% FCS/Penicillin-Streptomycin) and cultured at 37°C/5% CO_2_.

### Cytokine Measurement

Unfractionated wound cells, column-enriched monocytes/macrophages or FACS-sorted wound monocyte/macrophage populations were cultured in complete medium (DMEM/5% FCS/Penicillin-Streptomycin) or serum-free X-Vivo media (for the detection of TGF-β, Lonza) and cell supernatants removed after 24 h or 72 h for cytokine analysis. 72 h cell supernatants were tested by sandwich ELISA for TNF-α (BD Biosciences OptEIA mono/mono ELISA Set II), and TGF-β (DuoSet, R&D Systems). 24 h cell supernatants were tested by sandwich ELISA for IL-1β (BD Biosciences OptEIA mono/mono ELISA Set II) and VEGF (DuoSet, R&D Systems). Culture times were selected for optimal detection of cytokines in supernatants within the ELISA standard curves.

### *In vivo* Microparticle Labeling of Monocyte Subsets

Selective labeling of circulating monocyte subsets was performed with methods adapted from those described in Tacke *et al* [33]. To label Ly6C^hi^ monocytes, 250 µL of liposome-encapsulated clodronate (Dr. Nico van Rooijen; ClodoronateLiposomes.com) was administered i.v. to B6 mice to deplete circulating monocytes. 18 h after clodronate injection, mice received an i.v. injection of Fluoresbrite Polychromatic Red Microbeads (PE-MP; 0.2 µg per mouse, Polysciences, Inc.). PVA sponges were implanted 1 day after bead injection, harvested 1 or 7 days later and processed for cell surface staining as described above. Blood cells were also isolated from wounded animals as described and processed for cell surface staining. The presence of PE-microbeads in blood and wound monocyte/macrophage subsets was assessed by FACS.

To label Ly6C^low^ monocytes, PE-MP were administered i.v. without prior treatment with liposome-encapsulated clodronate. B6 mice received sponges 1 day after bead injection. Blood and sponge cells were harvested 1 or 7 days later and prepared for flow cytometry analysis as previously described.

### PVA Sponge Adoptive Transfer

CD45.1 congenic mice received sponges for 1 day. Sponges were then removed from CD45.1 congenic (donor) mice under sterile conditions and transferred to unwounded B6 (recipient) mice. Sponges were harvested from recipient mice at 1, 3 or 7 days post-transfer. Cells were isolated from sponges and surface stained as previously described. CD45.1^+^ and CD45.2^+^ wound monocyte/macrophage subsets were analyzed by FACS at indicated time points.

### Gene Expression Analysis of Macrophage Subsets

Macrophages from the day 14 wound were enriched through negative selection on a magnetic column (Miltenyi Biotec) using the following depletion cocktail: Ly6G-PE (clone 1A8, BD Biosciences), CD2-PE (clone RM2–5; BD Biosciences), Siglec-F-PE (clone E50–2440; BD Biosciences) and TER119/Erythroid Cells-PE (clone Ter-119; BD Biosciences). Staining and column enrichment were carried out as previously described. Following enrichment, wound macrophages were surface stained with Ly6C-FITC, Ly6G-PE, Siglec-F-PE and F4/80-APC for 30 minutes on ice. Sytox Blue (Invitrogen) was used for dead cell exclusion. Ly6C^hi^F4/80^+^Ly6G^–^Siglec-F^–^ and Ly6C^low/int^F4/80^+^Ly6G^–^Siglec-F^–^ subsets were sorted to ≥90% purity under sterile conditions using a FACSAria.

RNA was isolated from sorted cells using the RNeasy Micro Kit (Qiagen) and cDNA was synthesized with the High Capacity RNA-to-cDNA Kit (Life Technologies). Gene expression analysis was performed by real-time quantitative PCR on a ViiA 7 system (Life Technologies) using TaqMan Assays (Life Technologies) and TaqMan Gene Expression Master Mix (Life Technologies). TaqMan Assay IDs for individual genes are shown in Table S1. *Hprt* and *18 s* served as endogenous controls. Both reference genes produced similar results after normalization. Results using *Hprt* are shown here. Analysis was carried out using the 2^–ΔΔCt^ method.

### Statistical Analysis

Statistical analysis was performed using ANOVA or Mann-Whitney tests. Statistical significance was defined as a p value of <0.05.

## Results

### Time Dependent Accumulation of Monocyte/Macrophage Subsets in the Sterile Wound

**Figure 1:**
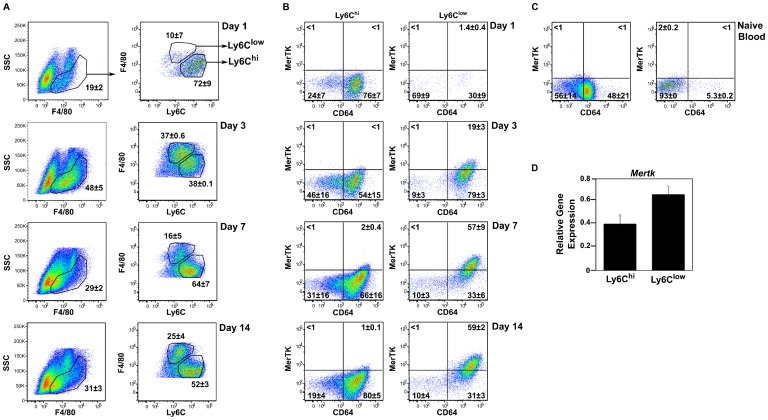
Phenotype of wound monocyte/macrophage subsets. C57BL/6J mice were wounded for 1, 3, 7 or 14 days. Wound cells were isolated and examined by flow cytometry. FSC and SSC were used to gate viable cells. (A) Wound monocytes/macrophages were defined as F4/80^+^SSC^low/int^. F4/80^int^SSC^hi^ eosinophils were excluded from analysis. Gated F4/80^+^ wound monocytes/macrophages were additionally examined for expression of Ly6C. (B) Expression of CD64 and MerTK was examined on gated Ly6C^hi^ and Ly6C^low^ monocyte/macrophage subsets. (C) Blood cells were isolated from naïve C57BL/6J mice and examined by flow cytometry. An identical gating strategy to that described for wound cells was employed to examine MerTK and CD64 expression on blood F4/80^+^Ly6C^hi^ and Ly6C^low^ monocytes. Numbers accompanying gates are the mean ± SD, n = 3 mice per group, and are representative of 2–3 independent experiments. (D) *Mertk* gene expression was determined in day 14 Ly6C^hi^ and Ly6C^low^ wound monocyte/macrophage subsets by qPCR. *Mertk* expression was normalized to *Hprt* and data are presented as ΔCt Expression. Data shown are the mean ± SD, n = 3 mice per group.

Differential analysis of cellular infiltrates in the sterile PVA sponge has demonstrated that monocytes are recruited to the wound within one day post-implantation, and that the frequency of monocytes/macrophages in the wound increases with time [34]. Distinct subsets of wound monocytes/macrophages could be distinguished by expression of the markers F4/80 and Ly6C. The day 1 F4/80^+^ wound cell population was predominantly Ly6C^hi/int^. The frequency, although not the absolute number, of this subset declined concurrent with the appearance of Ly6C^low/int^ cells by day 3 (Table S2). The proportion of Ly6C^hi^ cells increased at day 7 before falling slightly at day 14 (Figure 1A). By day 14, distinct F4/80^+^Ly6C^hi/int^ (hereafter Ly6C^hi^) and F4/80^hi^Ly6C^low/int^ (hereafter Ly6C^low^) subsets were apparent in the wound (Figure 1A).

**Figure 2:**
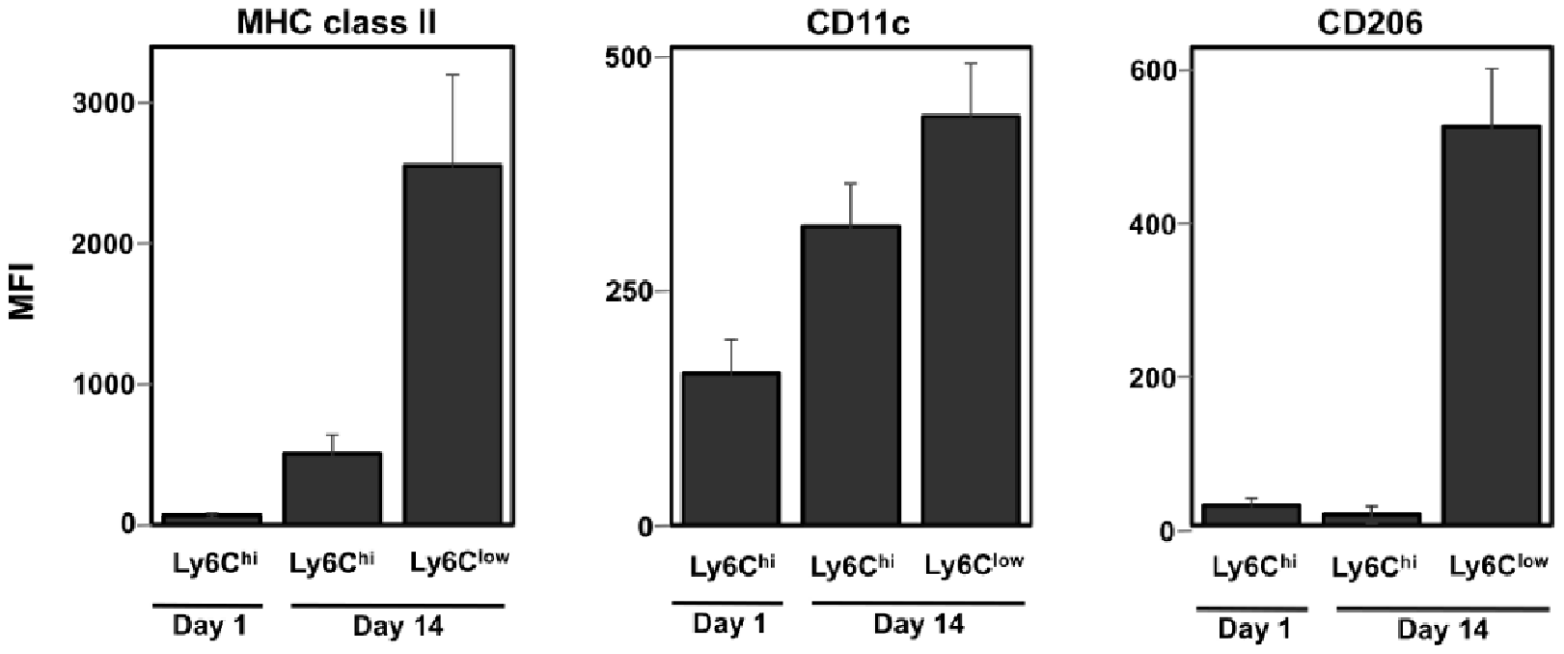
Surface marker expression on day 1 and day 14 wound macrophage subsets. C57BL/6J mice were wounded for 1 or 14 days, and the surface expression of MHC class II, CD11c and CD206 on wound monocyte/macrophage subsets was determined by flow cytometry. Wound monocyte/macrophage subsets were defined as F4/80^+^Ly6C^hi^ (Ly6C^hi^) and F4/80^+^Ly6C^low^ (Ly6C^low^). Mean fluorescence intensities (MFI; geometric mean) were calculated and are presented as the mean ± SD, n = 4 mice per group. Data are representative of at least 3 independent experiments.

Subsets were further examined for expression of CD64 and MerTK. These molecules, when co-expressed, have been identified as macrophage core markers [14], [15]. Ly6C^hi^ wound monocytes expressed CD64 at 1 (76±7%), 3 (54±15%), 7 (66±16%), and 14 (80±5%) days after injury. The frequency of MerTK expression by these cells at all examined time points was ≤2% (Figure 2B). This phenotype was similar to that of circulating Ly6C^hi^ monocytes, which were CD64^+^MerTK^–^ or CD64^–^MerTK^–^ (Figure 1C). In contrast, wound Ly6C^low^ cells were more likely to co-express CD64 and MerTK (19±3% of cells 3 days after injury, 57±9% by post-wound day 7, and 59±2% on day 14; Figure 1B). qPCR analysis similarly revealed a higher relative expression of *Mertk* in Ly6C^low^ macrophages when compared to Ly6C^hi^ monocytes/macrophages at day 14 after wounding (Figure 1D). Expression of TAM family members Axl and Tyro3 on wound monocyte/macrophage subsets could not be detected by flow cytometry (data not shown).

Further cell surface marker analysis revealed that the day 1 and day 14 Ly6C^hi^ subsets were CD11c^low/int^MHC class II^low^CD206^–^. The expression of CD11c and MHC class II was higher on day 14 wound Ly6C^hi^ cells when compared to their day 1 counterparts. The day 14 Ly6C^low^ subset was MHC class II^+^, CD11c^+^ and CD206^+^ (Figure 2). A similar pattern of surface marker expression to that seen at day 14 was detected on monocyte/macrophage subsets isolated from day 7 wounds. MHC class II, CD11c and CD206 were more highly expressed on day 7 Ly6C^low^ macrophages than on Ly6C^hi^ monocytes, although the difference between the two subsets was not as pronounced as that observed on cells from day 14 wounds (data not shown). Cell cycle analysis revealed no monocyte/macrophage proliferation in the day 1 or day 14 wound (data not shown).

### Ly6C^low^ Wound Macrophages Arise from Ly6C^hi^ Monocytes *in situ*

Four independent approaches were taken to examine the origin of monocytes/macrophages in the wound. These included the tracking of *in vivo*-labeled monocytes migrating to the wound, blood monocyte subset analysis before and after wounding, examination of chemokine receptor expression on blood and wound monocyte/macrophages subsets, and the adoptive transfer of wound sponges between CD45 congenic mice.

The monocytic origin of wound Ly6C^hi^ cells was first verified using the *in vivo* labeling techniques described by Tacke *et al* [33]. The i.v. injection of fluorescent microparticles (MP) to animals previously depleted of monocytes by the administration of liposome-encapsulated clodronate results in the selective labeling of F4/80^+^Ly6C^hi^ blood monocytes. In contrast, the MP are taken up almost exclusively by F4/80^+^Ly6C^low^ monocytes when injected into naïve mice. MP uptake has been demonstrated to not affect the recruitment of monocytes from the blood, nor to induce phenotypic markers of activation or the accumulation of proinflammatory cytokines in plasma. Further, no overt adverse effects of clodronate-mediated depletion were reported [33]. Indeed, this method of assessing monocyte trafficking presents advantages over adoptive transfer methods by eliminating the need for *ex vivo* cell manipulations that may alter the behavior of transferred cells, and provides greater sensitivity for quantification of migration [33].

**Figure 3:**
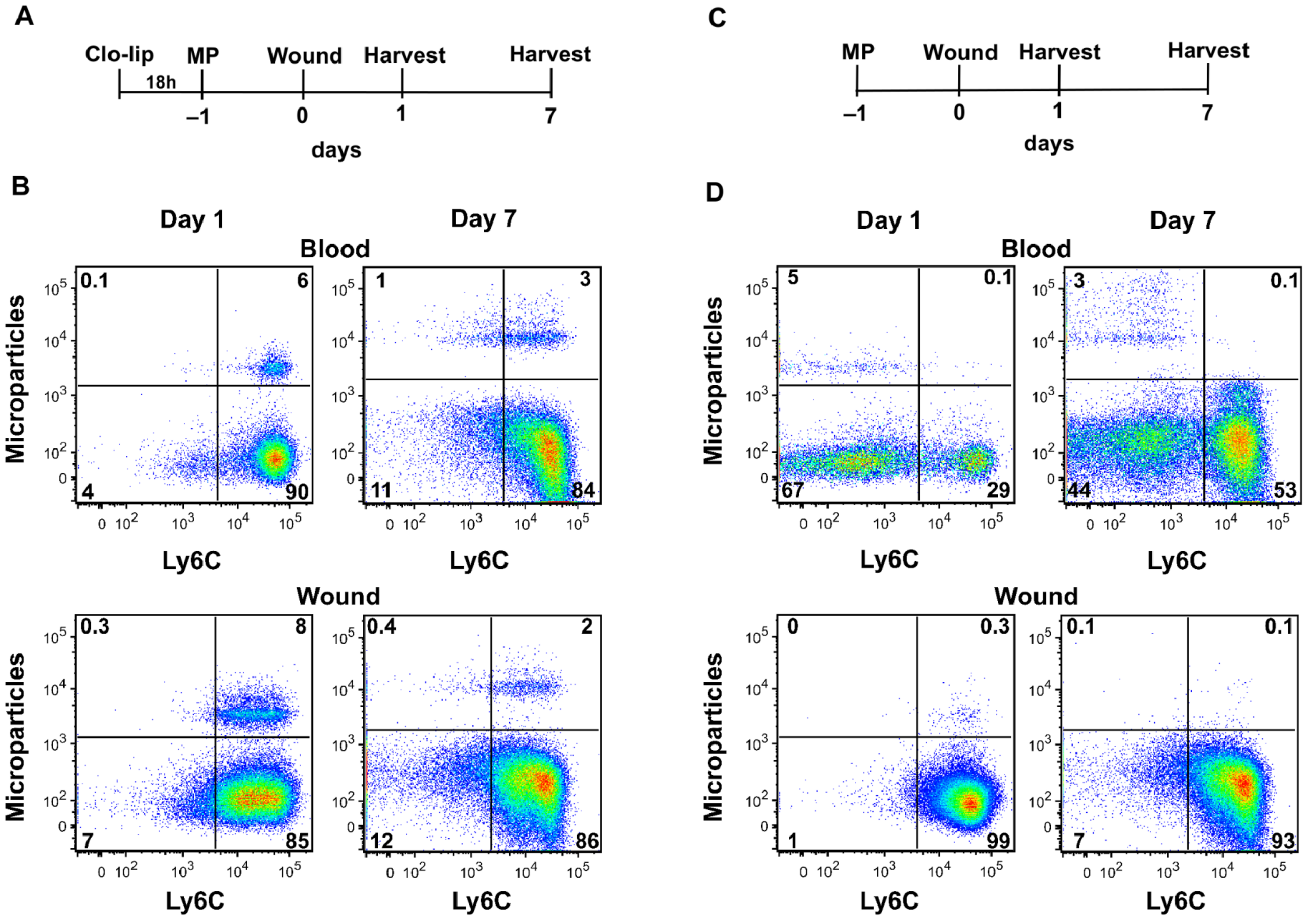
Migration of blood monocyte subsets to the wound. Ly6C^hi^ and Ly6C^low^ blood monocytes were selectively labeled with fluorescent microparticles (MP) following treatment with or without liposome-encapsulated clodronate (clo-lip) as described in Materials & Methods. C57BL/6J mice were then wounded for 1 or 7 days and accumulation of labeled monocytes/macrophages in the wound was assessed by flow cytometry. (A) Schematic of clo-lip and MP administration for the selective labeling of Ly6C^hi^ monocytes. Mice were wounded 1 day after MP delivery. (B) F4/80^+^ blood and wound cells were examined by flow cytometry following clodronate treatment and MP labeling. In blood and the day 1 and day 7 wound, Ly6C^hi^ monocytes were predominantly labeled with MPs. (C) Schematic of MP treatment for the selective labeling of circulating Ly6C^low^ monocytes prior to wounding for 1 or 7 days. (D) F4/80^+^ blood and wound cells were examined by flow cytometry following selective labeling of Ly6C^low^ monocytes. MP-labeled Ly6C^low^ monocytes were identified in the circulation but not in the day 1 or day 7 wound. The proportion of cells in quadrants is indicated by numbers on plots. N = 2 mice per group.

Figure 3A is a schematic demonstrating the order and timing of liposome-encapsulated clodronate and MP administration prior to wounding. In the blood, MP administered after liposome-encapsulated clodronate treatment were observed predominantly in the F4/80^+^Ly6C^hi^ subset at 1 and 7 days post-wounding, while approximately 1% of blood monocytes were MP^+^Ly6C^low^ at day 7 (Figure 3B). As expected, clodronate treatment resulted in a reduction of the Ly6C^low^ circulating monocyte population at days 1 and 7 after wounding (Figure 3B). However, this did not affect the pattern of wound monocyte/macrophage accumulation, as the distribution of Ly6C^hi^ and Ly6C^low^ wound monocytes/macrophages was similar with or without clodronate treatment (Figures 3B and 3D). MP-containing cells were detected in the day 1 and day 7 wound following selective labeling of Ly6C^hi^ blood monocytes. The majority of MP-labeled F4/80^+^ wound cells at both time points were Ly6C^hi^. A small proportion of the labeled monocytes at day 7 were Ly6C^low^, possibly due to maturation of Ly6C^hi^ cells in the wound or the migration of the small fraction of MP^+^Ly6C^low^ blood monocytes observed at this time point [1], [3], [35].

A similar tracking approach was adopted to examine whether circulating Ly6C^low^ monocytes were recruited to the wound (schematic shown in Figure 3C). After intravenous injection of labeled MP into naïve animals, F4/80^+^MP^+^ monocytes were detected in the blood after wounding, and nearly 100% of these cells were Ly6C^low^ (Figure 3D). Examination of wound cells at 1 and 7 days after sponge insertion failed to detect labeled Ly6C^low^ monocytes/macrophages among infiltrating F4/80^+^ cells (Figure 3D). Approximately 0.3% of wound monocytes were MP^+^Ly6C^hi^ at day 1, perhaps due to the migration of MP-labeled Ly6C^hi^ monocytes from the blood (0.1% MP^+^Ly6C^hi^, Figure 3D).

**Figure 4:**
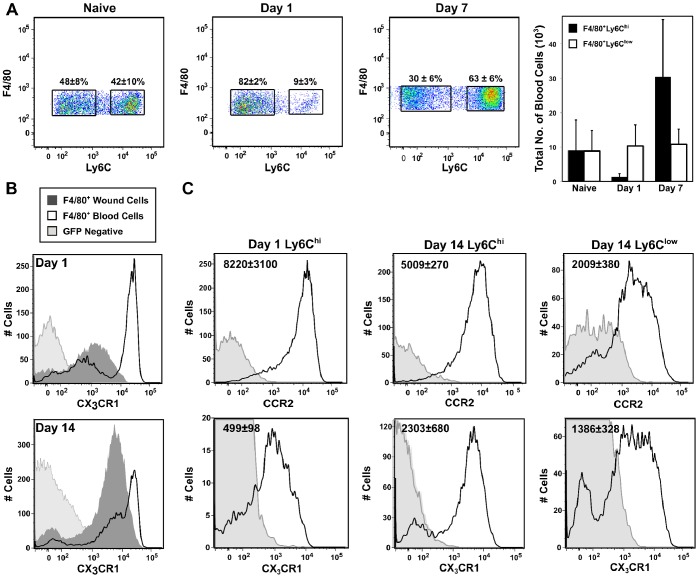
Chemokine receptor expression on circulating and wound monocytes/macrophages. (A) Blood cells were isolated from naïve, day 1 and day 7-wounded C57BL/6J mice. Gated F4/80^+^SSC^low^ blood monocytes were examined for Ly6C expression in naïve and day 1-wounded mice. Mean frequencies (±SD) are indicated above each gate. The mean number ± SD of F4/80^+^Ly6C^hi^ and Ly6C^low^ blood monocytes is also shown. (B) Expression of CX_3_CR1 was compared on circulating and wound F4/80^+^ monocyte/macrophage subsets at 1 and 14 days after wounding by flow cytometry using CX_3_CR1-GFP transgenic mice. (C) C57BL/6J or CX_3_CR1-GFP transgenic mice were wounded for 1 or 14 days. Gated F4/80^+^Ly6C^hi^ and Ly6C^low^ wound monocytes/macrophages were examined by flow cytometry for CCR2 and CX_3_CR1 expression. Average MFI ± SD (geometric mean) is indicated in each plot. N = 3–5 mice per group. Data are representative of at least 3 independent experiments.

It was further noted that Ly6C^hi^ but not Ly6C^low^ monocytes were transiently diminished in the circulation at 1 day after wounding, suggesting preferential trafficking of this subset to the wound (Figure 4A). The Ly6C^low^ monocyte count in the circulation remained constant over the time points examined (Figure 4A).

The pattern of chemokine receptor expression on circulating and wound monocytes/macrophages was also consistent with a monocytic origin for Ly6C^hi^ wound cells. CX_3_CR1 is highly expressed on circulating Ly6C^low^ monocytes and putatively involved in their recruitment to inflammatory sites [6]. CX_3_CR1^hi^ and CX_3_CR1^low^ monocytes were detected in the blood of transgenic mice expressing GFP under the control of the CX_3_CR1 promoter after wounding (Figure 4B). In contrast, wound monocytes/macrophages harvested 1 or 14 days after wounding were CX_3_CR1^low/int^ (Figure 4B). Ly6C^hi^ and Ly6C^low^ subsets at day 14 expressed similar levels of CX_3_CR1 (Figure 4C). Expression of CX_3_CR1 was moderate on day 14 wound cell subsets when compared to blood CX_3_CR1^hi^ monocytes, but higher than that seen on day 1 Ly6C^hi^ wound monocytes/macrophages (Figure 4C). Furthermore, in contrast to the inverse relationship between CX_3_CR1 and CCR2 expression on blood monocytes, day 1 and day 14 F4/80^+^ wound cells were found to co-express these chemokine receptors, regardless of Ly6C status (Figure 4C).

**Figure 5:**
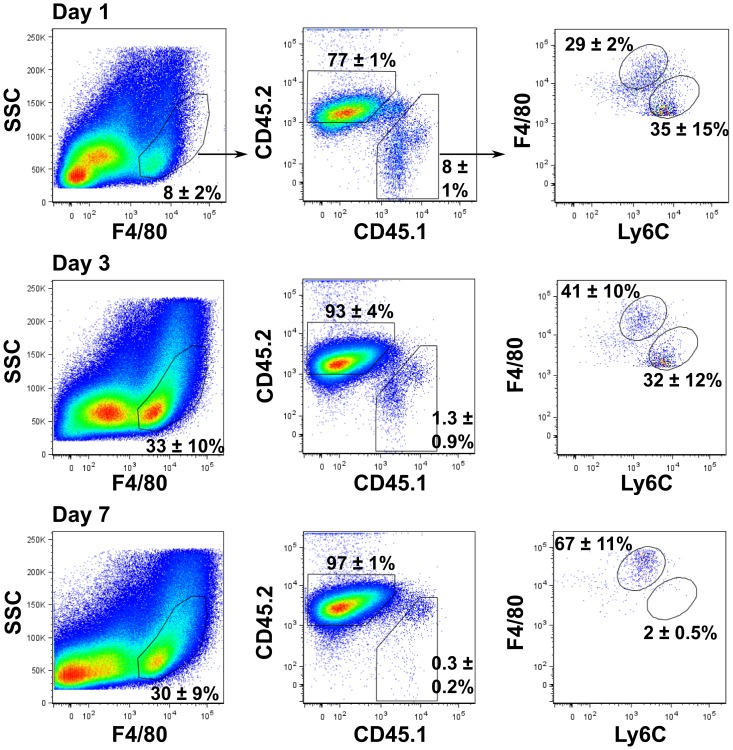
Ly6C^hi^ monocytes mature *in situ* into Ly6C^low^ wound macrophages. Sponges were implanted into CD45.1 congenic mice. At 1 day post-wounding, sponges were transferred from donor CD45.1 congenic mice to recipient B6 (CD45.2) mice as described in Materials & Methods. Wound cells were isolated from recipient mice at 1, 3 or 7 days post-transfer and monocyte/macrophage subsets were analyzed by flow cytometry. CD45.1^+^ cells were identified on gated F4/80^+^SSC^low/int^ cells and examined for F4/80^+^Ly6C^hi^ and Ly6C^low^ subsets. Numbers accompanying gates are the mean frequency ± SD, n = 3 mice per group. Data are representative of at least 2 independent experiments.

To examine the potential for Ly6C^hi^ wound monocytes to mature into Ly6C^low^ cells *in situ*, sponge adoptive transfer experiments were performed using mice expressing the congenic markers CD45.1 or CD45.2. Wound cells from congenic strains had comparable expression of F4/80 and Ly6C. Sponges were removed from CD45.1 donor mice 1 day after wounding when >70% of F4/80^+^ cells were Ly6C^hi^ and ≤10% were Ly6C^low^ (Figure 1A) and transferred to CD45.2^+^ recipients. Sponges were harvested at 1, 3, or 7 days post-transfer, and the phenotype of donor CD45.1^+^ cells was analyzed by flow cytometry (Figure 5). The frequency of donor-derived CD45.1^+^ cells decreased over time due to the accumulation of CD45.2^+^ recipient cells in the wound. However, a similar number of F4/80^+^CD45.1^+^ donor-derived cells were recovered at days 1 and 7 after transfer (Table S3). The presence of CD45.1^+^ donor-derived cells in the sponge did not affect the recruitment or phenotype of CD45.2^+^ recipient cells in the wound upon sponge transfer. Examination of donor-derived CD45.1^+^F4/80^+^ cells revealed a decrease in the proportion and total number of Ly6C^hi^ cells over time (Figure 5 and Table S3). At days 1 and 3 post-transfer, 35±15% and 32±12% of donor monocytes/macrophages, respectively, were Ly6C^hi^. At day 7, only 2±0.5% of donor-derived F4/80^+^ wound cells were Ly6C^hi^. In contrast, the frequency and total number of Ly6C^low^ donor-derived F4/80^+^ cells increased with time (Figure 5 and Table S3). At day 1, 29±2% of the F4/80^+^ population was Ly6C^low^. This proportion increased to 41±10% at day 3 and 67±11% at day 7. The recovery of a similar number of CD45.1^+^F4/80^+^ cells at 1 and 7 days post-transfer suggests that these results are not an artifact of selective survival of donor Ly6C^low^ cells, but rather due to *in situ* conversion of Ly6C^hi^ monocytes.

### Early Wound Monocytes Acquire Function in the Wound and are Distinct from Late Macrophages

**Figure 6:**
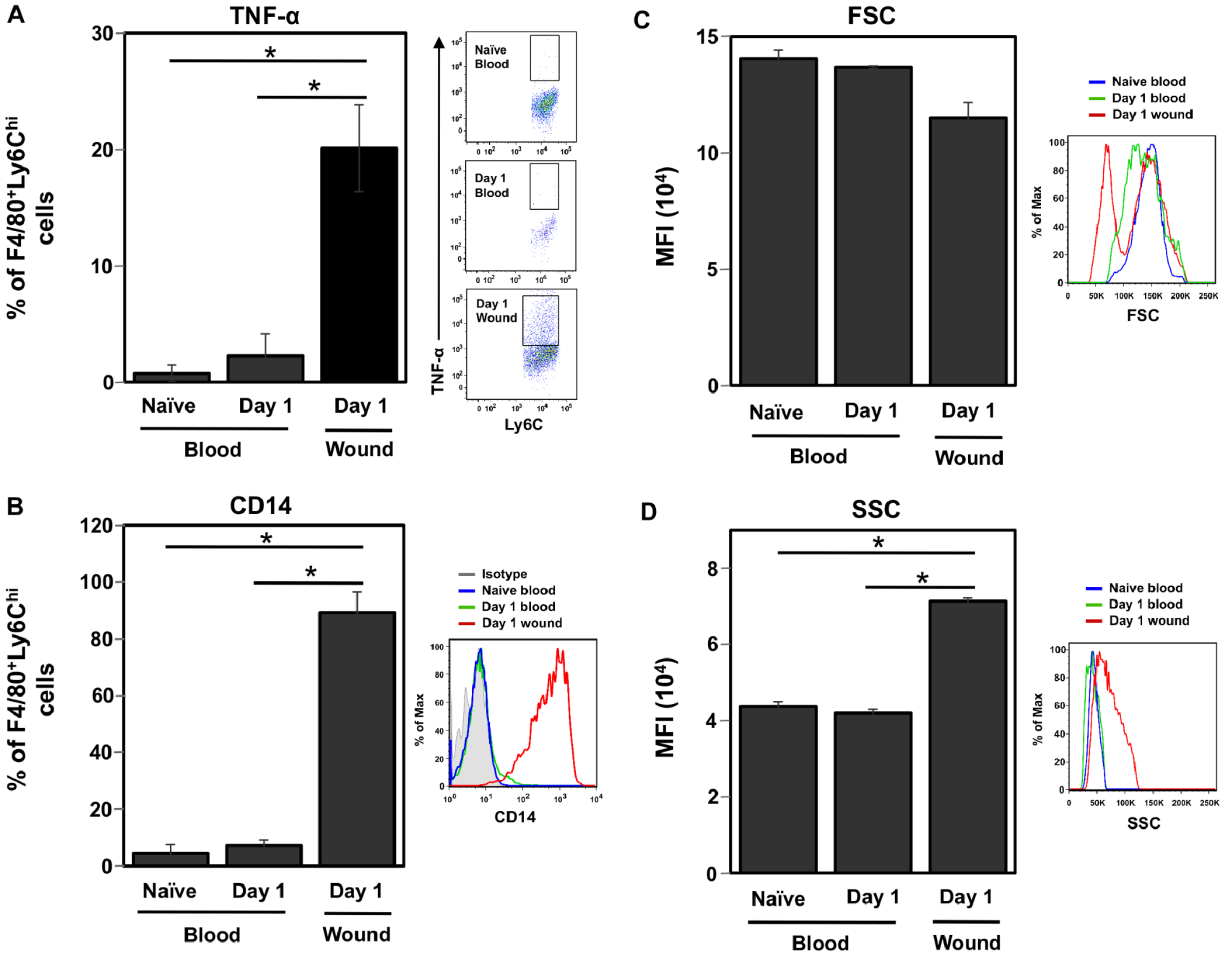
Changes in monocyte phenotype upon entry to the wound. Gated F4/80^+^Ly6C^hi^ blood monocytes from naïve and day 1-wounded C57BL/6J mice were compared to wound monocytes from day 1-wounded mice by flow cytometry analysis. (A) Proportion of TNF-α^+^ cells and representative FACS plots, (B) proportion of CD14^+^ cells and representative histograms, (C) FSC median fluorescence intensity (MFI) and (D) SSC MFI with representative histograms. Data shown are the mean ± SD, n = 3 mice per group for (A) and (B); n = 2–3 mice per group for (C) and (D). Data are representative of 2–3 independent experiments.

Ly6C^hi^ cells in blood and wound were compared to determine the acquisition of inflammatory traits upon entry to the wound. FACS analysis of intracellular TNF-α revealed that 20% of day 1 Ly6C^hi^ wound monocytes spontaneously expressed this cytokine (Figure 6A). This was in contrast to Ly6C^hi^ blood monocytes from naïve and day 1-wounded mice, where less than 5% of the population expressed TNF-α. CD14 was also upregulated upon entry to the wound. Circulating monocytes in naïve and day 1-wounded mice expressed low levels of CD14, while nearly 100% of Ly6C^hi^ monocytes in the day 1 wound were CD14^hi^ (Figure 6B). Additionally, analysis of forward scatter (FSC) and side scatter (SSC) properties revealed that Ly6C^hi^ monocytes in the day 1 wound had a similar FSC profile but higher SSC than circulating monocytes, suggesting that the cells gained greater internal complexity upon entry to the wound (Figure 6C and D).

**Figure 7:**
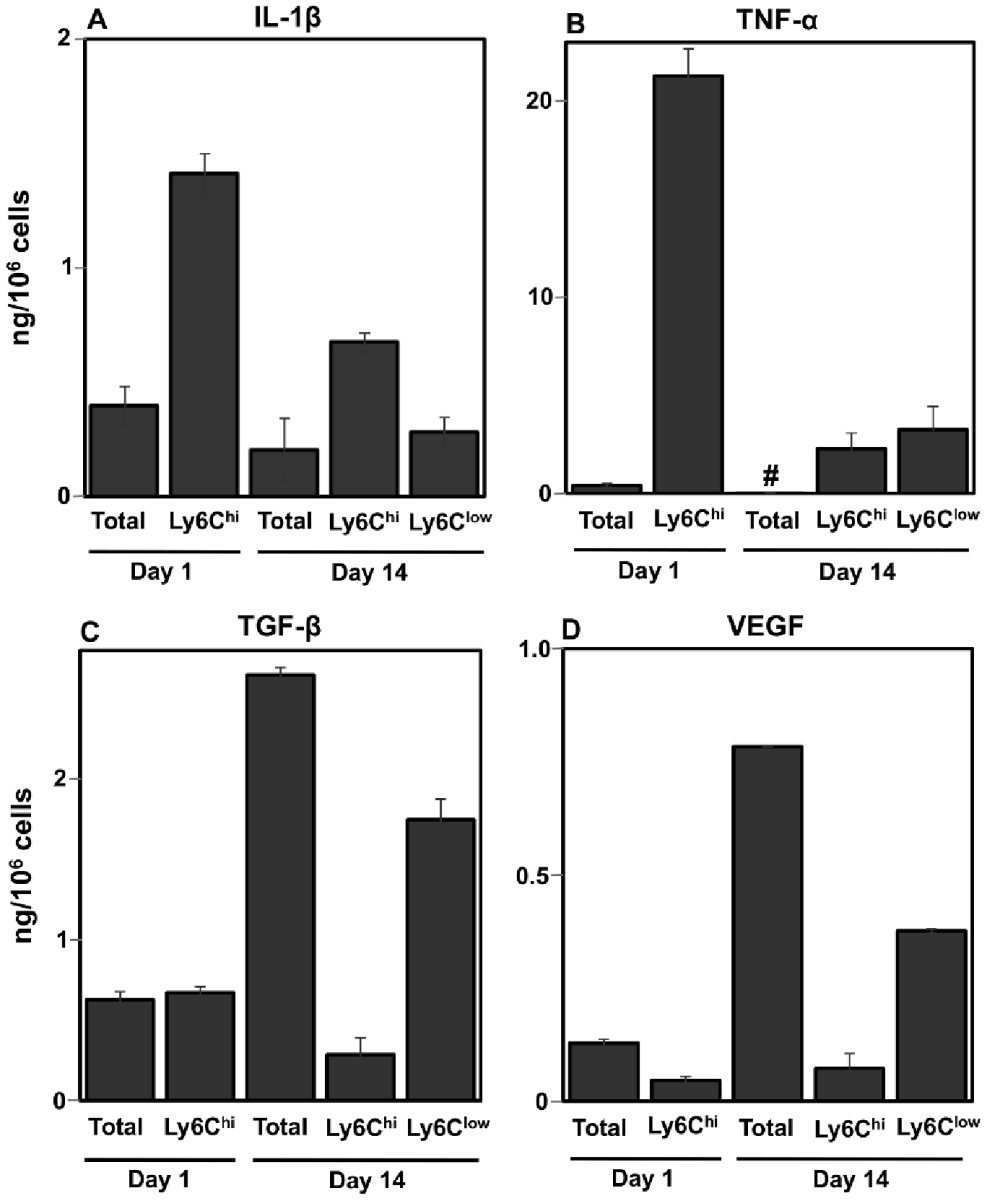
Cytokine production by wound monocyte/macrophage subsets. F4/80^+^Ly6C^hi^ monocytes/macrophages were sorted from day 1 wound cells. F4/80^+^Ly6C^hi^ and Ly6C^low^ monocytes/macrophages were sorted from day 14 wound cells. Sorted populations were cultured as described in Materials & Methods and supernatants were tested by ELISA for (A) IL-1β, (B) TNF-α, (C) TGF-β and (D) VEGF production. Data shown are the mean ± SD; n = 3 mice per experiment; data represent 2–3 independent experiments. # = below the level of detection.

The functional transition from early wound inflammatory monocytes to repair macrophages was examined by *ex vivo* cytokine production profiles (Figure 7). Day 1 and day 14 Ly6C^hi^ and day 14 Ly6C^low^ wound monocytes/macrophages were FACS sorted and cultured alongside unfractionated wound cells. Culture supernatants were tested by ELISA for production of proinflammatory and repair-associated cytokines. The highest levels of both IL-1β and TNF-α were detected in culture supernatants of Ly6C^hi^ cells enriched from day 1 wounds (Figure 7A and B). These cells produced 3.5-fold more IL-1β than unfractionated cells from day 1 wounds, 7-fold more than day 14 total cells, and 2- and 5-fold more than Ly6C^hi^ and Ly6C^low^ cells recovered from day 14 wounds, respectively (Figure 7A). TNF-α was abundant in cultures from isolated day 1 Ly6C^hi^ cells, in which the measured release was approximately 50-fold higher than that in cultures of day 1 unfractionated wound cells, and approximately 10-fold higher than in cultures from Ly6C^hi^ and Ly6C^low^ cells from the day 14 wound. TNF-α production by day 14 unfractionated wound cells was below the level of detection of the assay (Figure 7B).

The highest concentrations of TGF-β and VEGF were detected in day 14 wound cell cultures. A comparison of TGF-β production by day 14 wound monocyte/macrophage subsets revealed that Ly6C^low^ cells preferentially produced this cytokine, making 6-fold more than Ly6C^hi^ cells at the same time point (Figure 7C). Production of TGF-β by day 14 wound Ly6C^low^ cells was also higher than that by day 1 unfractionated and Ly6C^hi^ cells. Similarly, day 14 Ly6C^low^ cells produced over 5-fold more VEGF than their Ly6C^hi^ counterparts, and more than day 1 total and Ly6C^hi^ cells (Figure 7D). For both TGF-β and VEGF, the highest concentrations were detected in day 14 unfractionated cell cultures, indicating that monocytes/macrophages are not the exclusive source of these cytokines (Figure 7C and D).

Comparative gene expression analysis revealed relatively higher levels of *Il1b*, *Tnf*, *Tgfb* and *Vegf* cytokine message in the Ly6C^hi^ subset relative to the Ly6C^low^ subset. The difference between populations was most pronounced for *Il1b* expression, which was 36-fold higher in Ly6C^hi^ relative to Ly6C^low^ F4/80^+^ cells. The Ly6C^hi^ subset had an approximately 2-fold higher expression level of *Tnf, Vegfa* and *Tgfb1* in comparison to Ly6C^low^ cells (Figure S1).

### Gene Expression Analysis of Day 14 Wound Monocytes/Macrophages

**Figure 8:**
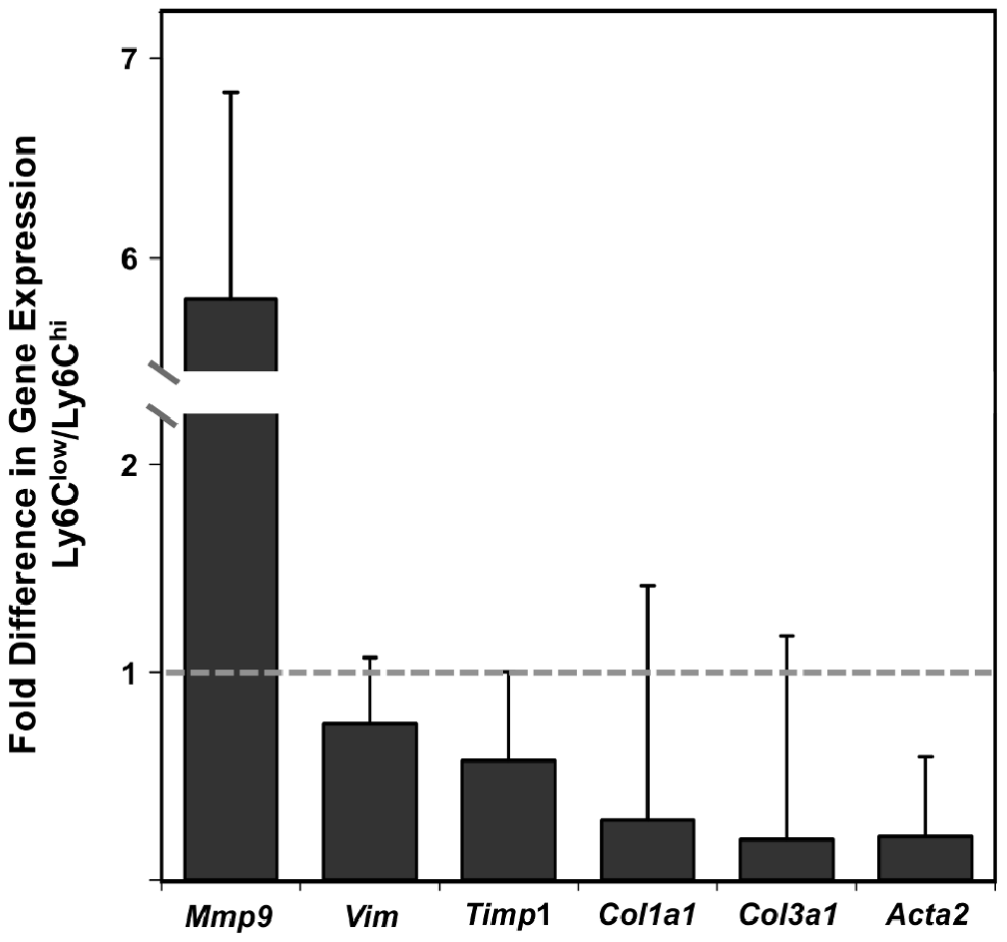
Comparative gene expression analysis of day 14 Ly6C^hi^ and Ly6C^low^ macrophages. F4/80^+^Ly6C^hi^ and F4/80^+^Ly6C^low^ macrophages were FACS-sorted from the day 14 wound. Expression of matrix metalloproteinase 9 (*Mmp9*), vimentin (*Vim*), tissue inhibitor of metalloproteinase 1 (*Timp1*), collagen 1 (*Col1a1*), collagen 3 (*Col3a1*) and α-smooth muscle actin (*Acta2*) was examined by qPCR. Target gene expression was normalized to *Hprt*. ΔΔCt Expression is presented as fold difference in relative gene expression of Ly6C^low^ relative to Ly6C^hi^ cells as described in Materials & Methods. Equal gene expression between subsets is indicated by a grey dashed line placed at a fold change of 1. Data are means ± SD, n = 3 mice per group.

Day 14 wound Ly6C^hi^ and Ly6C^low^ monocytes/macrophages were assessed for the differential expression of genes related to fibroblastic and/or mesenchymal transition. Ly6C^hi^ and Ly6C^low^ monocytes/macrophages were sorted from the day 14 wound, and relative expression of *matrix metalloproteinase 9* (*Mmp9*), *vimentin* (*Vim*), *tissue inhibitor of metalloproteinase 1* (*Timp1*), *type I collagen* (*Col1a1*), *type III collagen* (*Col3a1*), and *α-smooth muscle actin* (*Acta2*) was determined by qPCR (Figure 8). These genes were selected based on reports examining mesenchymal transition in myeloid cells [16]–[18]. Ly6C^low^ wound macrophages had approximately 6-fold higher levels of *Mmp9* expression relative to Ly6C^hi^ cells. In contrast, *Acta2* was expressed at lower levels in Ly6C^low^ macrophages. *Vim, Timp1, Col1a1* and *Col3a1* relative expression was approximately equal between both monocyte/macrophage subsets (Figure 8).

### MerTK Regulates the Cellularity but not the Cytokine Environment in 14 Day-old Wounds

The role of MerTK in shaping the monocyte/macrophage response after PVA sponge implantation was investigated using mice that are deficient in MerTK signaling (MerTK^−/−^). The day 14 wound was chosen for analysis because the highest level of MerTK expression was detected on Ly6C^low^ wound macrophages at this time point (Figure 1C). MerTK expression on wound macrophage subsets at day 14 post-sponge implantation was comparable between B6129SF2/J control and B6 mice, and undetectable by flow cytometry analysis on cells isolated from the wounds of MerTK^−/−^ mice (Figure 1B and data not shown).

**Figure 9:**
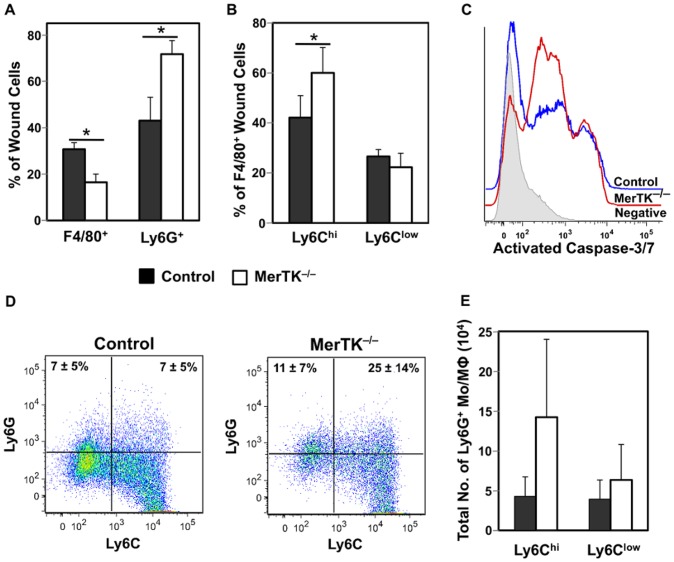
Cellular responses to sterile injury in MerTK^−/−^ mice. Control or MerTK^−/−^ mice received sponges for 14 days. (A) Flow cytometry analysis was used to determine the proportion of F4/80^+^ macrophages and Ly6G^+^ neutrophils in the day 14 wounds of control and MerTK^−/−^ mice. (B) The F4/80^+^ macrophage population was assessed for the frequency of Ly6C^hi^ and Ly6C^low^ subsets. (C) The expression of activated caspase-3/7 was determined by FACS in control (blue histogram) and MerTK^−/−^ (red histogram) mice at 14 days after wounding. The negative control is shown as a grey histogram. As an indication of neutrophil phagocytosis by wound monocytes/macrophages (Mo/Mφ), the frequency (D) and number (E) of Ly6C^hi^ and Ly6C^low^ cells expressing intracellular Ly6G was determined by FACS. Representative dot plots showing intracellular Ly6G expression in Ly6C^hi^ and Ly6C^low^ wound monocytes/macrophages are shown in (D). Plots were gated on F4/80^+^ cells. Proportion of Ly6C^hi^Ly6G^+^ and Ly6C^low^Ly6G^+^ cells (±SD) is indicated in quadrants. Data shown in (D) are representative of 6 mice. Data shown in A–D are the mean ± SD, n = 6 mice per group. * p<0.05.

MerTK deficiency did not alter the total number of wound cells recovered 14 days after injury (control, 5.1±2.7x10^6^ vs. MerTK^−/−^, 7.3±2.9x10^6^, n = 6, p>0.05). However, day 14 wounds from MerTK^−/−^ mice contained a greater frequency of Ly6G^+^ neutrophils and a lower proportion of F4/80^+^ cells than controls (Figure 9A). The F4/80^+^ population isolated from wounds in MerTK^−/−^ mice contained a higher frequency of Ly6C^hi^ cells when compared to controls (Figure 9B). Overall, the composition of cellular infiltrates in day 14 wounds from MerTK^−/−^ mice resembled that seen in less mature wounds in normal mice.

MerTK has been reported to function in the phagocytic clearance of apoptotic debris. However, the fraction of wound cells expressing activated caspase-3/7 in day 14 wounds was similar in MerTK^−/−^ and control mice (26% ±5 and 37% ±8, respectively, n = 6 mice; Figure 9C). Moreover, when day 14 wound macrophages from B6 mice were stained for intracellular Ly6G as a marker for phagocytosed neutrophils, Ly6C^hi^MerTK^–^ and Ly6C^low^MerTK^+^ wound monocyte/macrophage populations were comparable in their expression of this marker by both frequency (Figure 9D) and total number (Figure 9E).

Because MerTK activation has also been shown to modulate cytokine production by macrophages [26], [28], [36], TNF-α, VEGF and TGF-β concentrations were measured in fluids and enriched macrophage culture supernatants from day 14 wounds of control and MerTK^−/−^ mice. No differences were detected between groups (Table 1).

**Table 1:** Day 14 wound cytokines in MerTK^−/−^ and control mice.

Sample		TNF-α (pg)	VEGF (pg)	TGF-β (pg)
Wound fluid	Control	86±40	608±124	1029±143
	MerTK^−/−^	102±26	709±218	852±403
Mono/Mac	Control	690±40	1650±370	820±190
	MerTK^−/−^	701±35	1730±410	870±210

Mice were wounded for 14 days. Cytokine and growth factor concentrations were measured in wound fluids and monocyte/macrophage (Mono/Mac) culture supernatants. Data shown are means ± SD, n = 4–6 mice per group. Wound fluid cytokine concentrations are pg/mL; macrophage-secreted cytokines are pg/10^6^ cells.

## Discussion

Studies presented here were performed to examine the monocyte-to-macrophage transition in sterile wounds. Experiments were conducted using the subcutaneously implanted PVA sponge wound model. This model recapitulates the inflammatory, angiogenic, and fibrotic responses seen in all soft tissue wounds for at least two weeks following sponge implantation. Thereafter the continued fibrotic response to the sponge models for a sterile foreign body reaction. The PVA sponge wound model offers the singular advantage of allowing for the full recovery of viable infiltrating cells by simple mechanical disruption, without requiring the use of enzymatic digestion of the wound material. The biological response to the sponge does not depend upon an irritative effect of the implanted material, because an identical sequence of inflammation and fibrosis follows the subcutaneous insertion of sterile stainless steel mesh Hunt-Schilling chambers [37]. Importantly, the phenotype of macrophages isolated from the sponge wound has been shown to be the same as that of cells isolated from murine skin wounds [38].

Previous work with the PVA sponge model described the sequential accumulation of Gr-1^hi^ and Gr-1^low^ F4/80^+^ cells in the healing wound [34]. The addition of new phenotyping tools allowed for the identification of a subset of proinflammatory F4/80^+^Ly6C^hi^ monocytes and repair-like F4/80^+^Ly6C^low^ macrophages. Analysis of CD64 and MerTK expression on F4/80^+^ cells demonstrated that Ly6C^low^F4/80^hi^CD64^+^MerTK^+^ macrophages do not appear in the sterile wound immediately following injury. The early response was dominated by Ly6C^hi^F4/80^+^CD64^+^MerTK^–^ cells that phenotypically resembled circulating monocytes but expressed CD14 and had a proinflammatory signature including the ability to produce TNF-α and IL-1β. This observation was consistent with the report that alveolar monocytes expressed TNF-α and CD14 upon entry to the lung following CCL2-mediated inflammation, but otherwise resembled their circulating precursors [39].

A Ly6C^low/int^F4/80^+^CD64^+^ population appeared in the wound by 3 days post-injury and persisted through day 14. The proportion of Ly6C^low^ macrophages co-expressing CD64 and MerTK increased over time. This subset also expressed CD206 along with CD11c and MHC class II, and produced cytokines consistent with repair function (VEGF and TGF-β) [9], [34].

The cytokine profile of the Ly6C^low^ monocyte/macrophage subset suggested that these cells promote angiogenesis and fibrosis in the wound. Studies by others have proposed that macrophages exhibit functional and phenotypic plasticity with the ability to undergo transdifferentiation to fibroblast- or myofibroblast-like cells, which are essential to the fibrotic response. Multiple studies have described the upregulation of α-smooth muscle actin, a myofibroblast marker, on cells of myeloid origin during granulation tissue formation or in response to TGF-β [16]–[18]. Others found that Mac-1-expressing macrophages upregulated procollagen expression during *Schistosoma* infection, suggesting transdifferentiation into fibroblasts [40].

It is also possible that co-expression of myeloid and mesenchymal markers may result from the differentiation of a common precursor rather than lineage reprogramming. In this regard, fibrocytes are bone marrow-derived cells that co-express myeloid and hematopoietic markers, participate in fibrotic and angiogenic processes, and act as fibroblast and myofibroblast precurors [41]. Fibrocytes are characterized by their expression of the mesenchymal marker vimentin, and their ability to produce extracellular matrix proteins including collagens, as well as matrix remodeling enzymes, in particular MMP-9 [41]–[45]. It was hypothesized that the fate of the Ly6C^low^ wound macrophages described here may involve the acquisition of fibroblast or mesenchymal traits. However, qPCR analysis of collagens, α-smooth muscle actin, and vimentin did not reveal a subset-specific pattern of expression, at least at the singular time point examined here. Gene expression analysis did indicate a several-fold higher level of *Mmp9* in the Ly6C^low^ cells relative to the Ly6C^hi^ subset, while no difference was detected in *Timp1*. Despite this difference, the results do not support a fibrocyte origin or transdifferentiation program for the day 14 Ly6C^low^ wound macrophage. Ongoing studies will examine whether transdifferentiation is evident at other time points or by morphological analysis. While it is possible that transdifferentiation may result in loss of macrophage markers, evidence provided by Mooney *et al*. (2010) demonstrated that *csf1r-*EGFP cells recruited to a peritoneal foreign body implant acquired α-smooth muscle actin while retaining macrophage morphology and F4/80 expression, the marker used here for macrophage identification [16]. Experiments were performed 14 days after wounding because others have reported evidence for macrophage transdifferentiation at this time point in foreign body reactions in mice [16]. The findings reported here, however, do not rule out that mesenchymal transition may occur in wounds at a later time point.

The sequential nature of monocyte and macrophage accumulation in the wound led to the hypothesis that wound inflammatory monocytes derive from circulating monocytes that differentiate within the wound to become repair macrophages. Multiple approaches aimed at determining the origin and fate of Ly6C^hi^ wound monocytes/macrophages indicated that Ly6C^hi^ monocytes preferentially migrated from the circulation to the wound, and that this population changed *in situ* to Ly6C^low^ macrophages. These studies demonstrated that recruited inflammatory monocytes are precursors to repair macrophages in this model of sterile wound healing, which is consistent with the recruitment of F4/80^low^Ly6C^+^ proinflammatory monocytes and subsequent conversion to anti-inflammatory F4/80^hi^Ly6C^–^ monocytes/macrophages that occurs during skeletal muscle repair [5]. The selective migration of Ly6C^hi^ monocytes to the PVA sponge is in contrast to a model of myocardial infarction, in which sequential and independent recruitment of Ly6C^hi^ and Ly6C^low^ monocytes to the injury site was reported [6].

Within 3 days, a Ly6C^low/int^ population of F4/80^+^ cells appeared in the wound. This population was heterogeneous for MerTK expression, suggesting that it had not fully acquired a macrophage phenotype and, given the evidence for *in situ* maturation, may exist as an intermediate between Ly6C^hi^ inflammatory wound monocytes and Ly6C^low^ macrophages. The proportion of Ly6C^low^ macrophages expressing MerTK increased over time, reaching 59±2% by day 14 after wounding, and the level of MerTK expression (as measured by MFI) was higher on Ly6C^low^ than on Ly6C^hi^ cells at all time points examined (data not shown). Of note, the MerTK^+^F4/80^+^Ly6C^low^ population detected at day 14 after injury resembled a recently described human anti-inflammatory macrophage population that preferentially expresses MerTK and CD206 and possesses the ability to clear early apoptotic cells [20].

The kinetics and subset-specific MerTK expression on wound monocyte/macrophage populations raised the question of whether this kinase is involved in development of the repair macrophage phenotype. The reported functions of MerTK include phagocytosis of apoptotic cells and dampening production of proinflammatory mediators [19], [20]–[24], [26], [29], [46]. Accordingly, mice that lack MerTK are prone to LPS-induced endotoxic shock and the development of autoimmune disorders [21], [25], [27], [28]. Differential analysis has shown that neutrophils dominate the early wound, and that these cells persist even as monocytes/macrophages accumulate [32]. Work from this laboratory previously demonstrated that soluble products released from murine wound neutrophils dampen LPS-induced macrophage inflammatory responses *in vitro* [32]. Furthermore, using the PVA sponge model in rats, wound macrophages were found to both induce apoptosis and ingest wound neutrophils [30], [31]. It was hypothesized that MerTK may mediate some of these processes in the sterile wound and, as a result, promote the transition from inflammatory monocyte to repair macrophage.

The composition of inflammatory infiltrates from the wounds of MerTK^−/−^ mice was more immature than that from controls. Day 14 wounds from MerTK^−/−^ animals contained a higher proportion of neutrophils and F4/80^+^Ly6C^hi^ monocytes. This alteration in the wound cellularity, however, did not impact the cytokine and growth factor composition of wound fluids or monocyte/macrophage culture supernatants.

Contrary to what was expected given the known role of MerTK as a phagocytic receptor for apoptotic cells, there was no difference between MerTK-deficient and control mice in the number of wound cells expressing activated caspase-3/7, or in the frequency of wound macrophages that had ingested neutrophils. In further support of MerTK-independent phagocytosis, both Ly6C^hi^MerTK^–^ and Ly6C^low^MerTK^+^ wound monocytes/macrophages showed evidence of neutrophil ingestion *in vivo*.

While studies here did not reveal a difference in the wound environment beyond small changes in leukocyte populations after PVA sponge implantation in MerTK^−/−^ mice, a recent report found that MerTK-deficiency during myocardial infarction (MI) resulted in accumulation of apoptotic cardiomyocytes and subsequent adverse outcome [24]. Similar to what is reported here, this study found that MerTK was expressed predominantly on Ly6C^low^ macrophages in the myocardium after MI. Using bone marrow-derived macrophages, MerTK was found to promote the ingestion of apoptotic cardiomyocytes *in vitro*; it was not established, however, whether macrophages directly isolated from the healing myocardium were similarly MerTK-dependent for cardiomyocte phagocytosis. Furthermore, as indicated from studies here, Wan *et al*. did not link MerTK-deficiency to reduced clearance of neutrophils. This suggests that MerTK may function *in vivo* in the clearance of parenchymal, but not inflammatory cells. As the PVA sponge wound model lacks a site-specific parenchymal cell type [9], this observation may explain the lack of overt phenotype seen in MerTK^−/−^ mice. These data support the existence of alternative pathways for the clearance of apoptotic cells and modulation of cytokine production in this model of sterile injury, as has been reported elsewhere [47], [48].

Taken together, these studies more thoroughly define time-dependent accumulation of monocyte and macrophage subsets in the healing wound. The data revealed the persistence of Ly6C^hi^ monocytes in an inflamed sterile environment. While these cells expressed low to intermediate levels of CD11c and MHC class II, they were not TNF/iNOS-producing DCs (Tip-DCs), given that inflammatory cells do not express iNOS protein in the sterile wounds of mice [34], [49]. The finding of monocyte persistence in the wound is congruent with recent work by Jakubzick *et al.* [14], which reported that blood monocytes entering multiple tissue and lymph node sites under steady state conditions retained their monocyte status without acquiring macrophage or dendritic cell properties. The authors also proposed that Ly6C expression could provide a molecular signature for the identification of tissue monocytes. In the experiments presented here, however, it was noted that F4/80^+^Ly6C^low^ cells were heterogeneous for the expression of the macrophage-specific marker MerTK. This raises the question of whether Ly6C^low^MerTK^–^ cells in the sterile wound are true monocytes or macrophages, or represent a transitional phenotype.

Studies described here indicate that monocytes recruited to a sterile wound can undergo eventual, but not immediate, transition to macrophages with a reparative phenotype, which neither show proliferative capacity nor markers associated with transdifferentiation. The factors regulating the acquisition of inflammatory traits by monocytes upon entry to the sterile wound, as well as those that promote their development into repair macrophages, are unclear and are the subject of ongoing investigation.

## Supporting Information

Figure S1 **Relative expression of cytokine genes in monocyte/macrophage subsets.** Expression of IL-1β (*Il1b*), TNF-α (*Tnf*), TGF-β (*Tgfb1*) and VEGF (*Vegfa*) was determined from FACS-sorted day 14 Ly6C^hi^ and Ly6C^low^ wound monocytes/macrophages by qPCR. Data are shown as the ratio of gene expression in Ly6C^hi^ cells relative to Ly6C^low^ cells. A dashed grey line placed at a fold change of 1 is indicative of equal expression between subsets. Data shown are the mean ± SD, n = 3 mice per group.
(PDF)

Table S1 **TaqMan Assays Used in qPCR Experiments.**
(DOCX)

Table S2 **Number of wound monocyte/macrophage subsets in the wound.**
(DOCX)

Table S3 **Donor-derived cells recovered after sponge adoptive transfer.**
(DOCX)

## References

[1] Geissmann F, Jung S, Littman DR (2003) Blood monocytes consist of two principal subsets with distinct migratory properties. Immunity 19: 71–82. 12871640 10.1016/s1074-7613(03)00174-2

[2] Gordon S, Taylor PR (2005) Monocyte and macrophage heterogeneity. Nat Rev Immunol 5: 953–964 10.1038/nri1733 16322748

[3] Auffray C, Sieweke MH, Geissmann F (2009) Blood monocytes: development, heterogeneity, and relationship with dendritic cells. Ann Rev Immunol 27: 669–692 10.1146/annurev.immunol.021908.132557 19132917

[4] Geissmann F, Gordon S, Hume DA, Mowat AM, Randolph GJ (2010) Unravelling mononuclear phagocyte heterogeneity. Nat Rev Immunol 10: 453–460 10.1038/nri2784 20467425 PMC3032581

[5] Arnold L, Henry A, Poron F, Baba-Amer Y, van Rooijen N, et al (2007) Inflammatory monocytes recruited after skeletal muscle injury switch into antiinflammatory macrophages to support myogenesis. J Exp Med 204: 1057–1069 10.1084/jem.20070075 17485518 PMC2118577

[6] Nahrendorf M, Swirski FK, Aikawa E, Stangenberg L, Wurdinger T, et al (2007) The healing myocardium sequentially mobilizes two monocyte subsets with divergent and complementary functions. J Exp Med 204: 3037–3047 10.1084/jem.20070885 18025128 PMC2118517

[7] Ishida Y, Gao J-L, Murphy PM (2008) Chemokine receptor CX3CR1 mediates skin wound healing by promoting macrophage and fibroblast accumulation and function. J Immunol 180: 569–579. 18097059 10.4049/jimmunol.180.1.569

[8] Li L, Huang L, Sung S-SJ, Vergis AL, Rosin DL, et al (2008) The chemokine receptors CCR2 and CX3CR1 mediate monocyte/macrophage trafficking in kidney ischemia-reperfusion injury. Kidney Int 74: 1526–1537 10.1038/ki.2008.500 18843253 PMC2652647

[9] Brancato SK, Albina JE (2011) Wound macrophages as key regulators of repair: origin, phenotype, and function. Am J Pathol 178: 19–25 10.1016/j.ajpath.2010.08.003 21224038 PMC3069845

[10] Duffield JS, Forbes SJ, Constandinou CM, Clay S, Partolina M, et al (2005) Selective depletion of macrophages reveals distinct, opposing roles during liver injury and repair. J Clin Invest 115: 56–65 10.1172/JCI22675 15630444 PMC539199

[11] Goren I, Allmann N, Yogev N, Schürmann C, Linke A, et al (2009) A transgenic mouse model of inducible macrophage depletion: effects of diphtheria toxin-driven lysozyme M-specific cell lineage ablation on wound inflammatory, angiogenic, and contractive processes. Am J Pathol 175: 132–147 10.2353/ajpath.2009.081002 19528348 PMC2708801

[12] Lucas T, Waisman A, Ranjan R, Roes J, Krieg T, et al (2010) Differential roles of macrophages in diverse phases of skin repair. J Immunol 184: 3964–3977 10.4049/jimmunol.0903356 20176743

[13] Mirza R, DiPietro LA, Koh TJ (2009) Selective and specific macrophage ablation is detrimental to wound healing in mice. Am J Pathol 175: 2454–2462 10.2353/ajpath.2009.090248 19850888 PMC2789630

[14] Jakubzick C, Gautier EL, Gibbings SL, Sojka DK, Schlitzer A, et al. (2013) Minimal differentiation of classical monocytes as they survey steady-state tissues and transport antigen to lymph nodes. Immunity: 1–12. 10.1016/j.immuni.2013.08.007 PMC3820017 24012416

[15] Gautier EL, Shay T, Miller J, Greter M, Jakubzick C, et al (2012) Gene-expression profiles and transcriptional regulatory pathways that underlie the identity and diversity of mouse tissue macrophages. Nat Immunol 13: 1118–1128 10.1038/ni.2419 23023392 PMC3558276

[16] Mooney JE, Rolfe BE, Osborne GW, Sester DP, van Rooijen N, et al (2010) Cellular plasticity of inflammatory myeloid cells in the peritoneal foreign body response. Am J Pathol 176: 369–380 10.2353/ajpath.2010.090545 20008135 PMC2797897

[17] Jabs A, Moncada GA, Nichols CE, Waller EK, Wilcox JN (2005) Peripheral blood mononuclear cells acquire myofibroblast characteristics in granulation tissue. J Vasc Res 42: 174–180 10.1159/000084406 15767764

[18] Ninomiya K, Takahashi A, Fujioka Y, Ishikawa Y, Yokoyama M (2006) Transforming growth factor-beta signaling enhances transdifferentiation of macrophages into smooth muscle-like cells. Hypertens Res 29: 269–276 10.1291/hypres.29.269 16778334

[19] Seitz HM, Camenisch TD, Lemke G, Earp HS, Matsushima GK (2007) Macrophages and dendritic cells use different Axl/Mertk/Tyro3 receptors in clearance of apoptotic cells. J Immunol 178: 5635–5642. 17442946 10.4049/jimmunol.178.9.5635

[20] Zizzo G, Hilliard BA, Monestier M, Cohen PL (2012) Efficient clearance of early apoptotic cells by human macrophages requires M2c polarization and MerTK induction. J Immunol 189: 3508–3520 10.4049/jimmunol.1200662 22942426 PMC3465703

[21] Scott RS, McMahon EJ, Pop SM, Reap EA, Caricchio R, et al (2001) Phagocytosis and clearance of apoptotic cells is mediated by MER. Nature 411: 207–211 10.1038/35075603 11346799

[22] Sather S, Kenyon KD, Lefkowitz JB, Liang X, Varnum BC, et al (2007) A soluble form of the Mer receptor tyrosine kinase inhibits macrophage clearance of apoptotic cells and platelet aggregation. Blood 109: 1026–1033 10.1182/blood-2006-05-021634 17047157 PMC1785151

[23] Eken C, Martin PJ, Sadallah S, Treves S, Schaller M, et al (2010) Ectosomes released by polymorphonuclear neutrophils induce a MerTK-dependent anti-inflammatory pathway in macrophages. J Biol Chem 285: 39914–39921 10.1074/jbc.M110.126748 20959443 PMC3000973

[24] Wan E, Yeap X-Y, Dehn S, Terry RL, Novak ML, et al (2013) Enhanced efferocytosis of apoptotic cardiomyocytes through MER tyrosine kinase links acute inflammation resolution to cardiac repair after infarction. Circ Res 113: 1004–1012 10.1161/CIRCRESAHA.113.301198 23836795 PMC3840464

[25] Wallet MA, Sen P, Flores RR, Wang Y, Yi Z, et al (2008) MerTK is required for apoptotic cell-induced T cell tolerance. J Exp Med 205: 219–232 10.1084/jem.20062293 18195070 PMC2234377

[26] Lee Y-J, Han J-Y, Byun J, Park H-J, Park E-M, et al (2012) Inhibiting Mer receptor tyrosine kinase suppresses STAT1, SOCS1/3, and NF-κB activation and enhances inflammatory responses in lipopolysaccharide-induced acute lung injury. J Leukoc Biol 91: 921–932 10.1189/jlb.0611289 22427680

[27] Lu Q, Lemke G (2001) Homeostatic regulation of the immune system by receptor tyrosine kinases of the Tyro 3 family. Science 293: 306–311 10.1126/science.1061663 11452127

[28] Camenisch TD, Koller BH, Earp HS, Matsushima GK (1999) A novel receptor tyrosine kinase, Mer, inhibits TNF-alpha production and lipopolysaccharide-induced endotoxic shock. J Immunol 162: 3498–3503. 10092806

[29] Choi J-Y, Park H-J, Lee Y-J, Byun J, Youn Y-S, et al (2013) Upregulation of Mer receptor tyrosine kinase signaling attenuated lipopolysaccharide-induced lung inflammation. J Pharmacol Exp Ther 344: 447–458 10.1124/jpet.112.199778 23197771

[30] Meszaros AJ, Reichner JS, Albina JE (1999) Macrophage phagocytosis of wound neutrophils. J Leukoc Bio 65: 35–42. 9886244 10.1002/jlb.65.1.35

[31] Meszaros AJ, Reichner JS, Albina JE (2000) Macrophage-induced neutrophil apoptosis. J Immunol 165: 435–441. 10861082 10.4049/jimmunol.165.1.435

[32] Daley JM, Reichner JS, Mahoney EJ, Manfield L, Henry WL, et al (2005) Modulation of macrophage phenotype by soluble product(s) released from neutrophils. J Immunol 174: 2265–2272. 15699161 10.4049/jimmunol.174.4.2265

[33] Tacke F, Alvarez D, Kaplan TJ, Jakubzick C, Spanbroek R, et al (2007) Monocyte subsets differentially employ CCR2, CCR5, and CX3CR1 to accumulate within atherosclerotic plaques. J Clin Invest 117: 185–194 10.1172/JCI28549 17200718 PMC1716202

[34] Daley JM, Brancato SK, Thomay AA, Reichner JS, Albina JE (2010) The phenotype of murine wound macrophages. J Leukoc Bio 87: 59–67 10.1189/jlb.0409236 20052800 PMC2801619

[35] Sunderkötter C, Nikolic T, Dillon MJ, Van Rooijen N, Stehling M, et al (2004) Subpopulations of mouse blood monocytes differ in maturation stage and inflammatory response. J Immunol 172: 4410–4417. 15034056 10.4049/jimmunol.172.7.4410

[36] Fraineau S, Monvoisin A, Clarhaut J, Talbot J, Simonneau C, et al (2012) The vitamin K-dependent anticoagulant factor, protein S, inhibits multiple VEGF-A-induced angiogenesis events in a Mer- and SHP2-dependent manner. Blood 120: 5073–5083 10.1182/blood-201205429183 23065156

[37] Hunt TK, Jawetz E, Hutchison JG, Dunphy JE (1967) A new model for the study of wound infection. J Trauma 7: 298–306. 4959944 10.1097/00005373-196703000-00012

[38] Mirza R, Koh TJ (2011) Dysregulation of monocyte/macrophage phenotype in wounds of diabetic mice. Cytokine 56: 256–264 10.1016/j.cyto.2011.06.016 21803601

[39] Maus U, Herold S, Muth H, Maus R, Ermert L, et al (2001) Monocytes recruited into the alveolar air space of mice show a monocytic phenotype but upregulate CD14. Am J Physiol Lung Cell Mol Physio 280: L58–68. 10.1152/ajplung.2001.280.1.L58 11133495

[40] Bertrand S, Godoy M, Semal P, Van Gansen P (1992) Transdifferentiation of macrophages into fibroblasts as a result of Schistosoma mansoni infection. Int J Dev Biol 36: 179–184. 1445570

[41] Bellini A, Mattoli S (2007) The role of the fibrocyte, a bone marrow-derived mesenchymal progenitor, in reactive and reparative fibroses. Lab Invest 87: 858–870 10.1038/labinvest.3700654 17607298

[42] Bucala R, Spiegel LA, Chesney J, Hogan M, Cerami A (1994) Circulating fibrocytes define a new leukocyte subpopulation that mediates tissue repair. Mol Med 1: 71–81. 8790603 PMC2229929

[43] Blakaj A, Bucala R (2012) Fibrocytes in health and disease. Fibrogenesis Tissue Repair (Suppl 1): S6. doi:10.1186/1755-1536-5-S1-S6. 10.1186/1755-1536-5-S1-S6 PMC3368795 23259722

[44] Schmidt M, Sun G, Stacey MA, Mori L, Mattoli S (2003) Identification of circulating fibrocytes as precursors of bronchial myofibroblasts in asthma. J Immunol 171: 380–389. 12817021 10.4049/jimmunol.171.1.380

[45] Hartlapp I, Abe R, Saeed RW, Peng T, Voelter W, et al (2001) Fibrocytes induce an angiogenic phenotype in cultured endothelial cells and promote angiogenesis in vivo. FASEB J 15: 2215–2224 10.1096/fj.01-0049com 11641248

[46] Lee Y-J, Lee S-H, Youn Y-S, Choi J-Y, Song K-S, et al (2012) Preventing cleavage of Mer promotes efferocytosis and suppresses acute lung injury in bleomycin treated mice. Toxicol Appl Pharmacol 263: 61–72 10.1016/j.taap.2012.05.024 22687607

[47] Hart SP, Rossi AG, Haslett C, Dransfield I (2012) Characterization of the effects of cross-linking of macrophage CD44 associated with increased phagocytosis of apoptotic PMN. PloS One 7: e33142 10.1371/journal.pone.0033142 22427969 PMC3302854

[48] Eken C, Sadallah S, Martin PJ, Treves S, Schifferli JA (2013) Ectosomes of polymorphonuclear neutrophils activate multiple signaling pathways in macrophages. Immunobiology 218: 382–392 10.1016/j.imbio.2012.05.021 22749214

[49] Mahoney E, Reichner J, Bostom LR, Mastrofrancesco B, Henry W, et al (2002) Bacterial colonization and the expression of inducible nitric oxide synthase in murine wounds. Am J Pathol 161: 2143–2152. 12466130 10.1016/s0002-9440(10)64492-6 PMC1850895

