# Supplementary material for: The Monocyte to Macrophage Transition in the Murine Sterile Wound
Source: PLoS One. 2014 Jan 22;9(1):e86660. doi: 10.1371/journal.pone.0086660 (PMC3899284; doi:10.1371/journal.pone.0086660)
Supplement: Figure S1 — Relative expression of cytokine genes in monocyte/macrophage subsets. Expression of IL-1β (Il1b), TNF-α (Tnf), TGF-β (Tgfb1) and VEGF (Vegfa) was determined from FACS-sorted day 14 Ly6Chi and Ly6Clow wound monocytes/macrophages by qPCR. Data are shown as the ratio of gene expression in Ly6Chi cells relative to Ly6Clow cells. A dashed grey line placed at a fold change of 1 is indicative of equal expression between subsets. Data shown are the mean ± SD, n = 3 mice per group. (PDF) [file pone.0086660.s001.pdf]

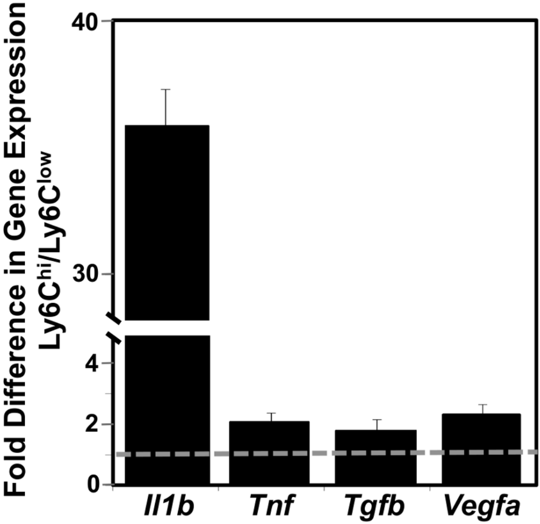

Figure S1. Relative expression of cytokine genes in monocyte/macrophage subsets. Expression of IL-1β (*Il1b*), TNF-α (*Tnf*), TGF-β (*Tgfb1*) and VEGF (*Vegfa*) was determined from FACS-sorted day 14 Ly6C^{hi} and Ly6C^{low} wound monocytes/macrophages by qPCR. Data are shown as the ratio of gene expression in Ly6C^{hi} cells relative to Ly6C^{low} cells. A dashed grey line placed at a fold change of 1 is indicative of equal expression between subsets. Data shown are the mean ± SD, n = 3 mice per group.
