# Supplementary material for: The Monocyte to Macrophage Transition in the Murine Sterile Wound
Source: PLoS One. 2014 Jan 22;9(1):e86660. doi: 10.1371/journal.pone.0086660 (PMC3899284; doi:10.1371/journal.pone.0086660)
Supplement: Table S1 — TaqMan Assays Used in qPCR Experiments. (DOCX) [file pone.0086660.s002.docx]

Table S1. TaqMan Assays Used in qPCR Experiments

| Gene | Assay ID |
| --- | --- |
| *Hprt* | Mm01545399_m1 |
| *18s* | Hs99999901_s1 |
| *Il1b* | Mm00434228_m1 |
| *Tnf* | Mm00443260_g1 |
| *Tgfb1* | Mm01178820_m1 |
| *Vegfa* | Mm01281449_m1 |
| *Col1a1* | Mm00801666_g1 |
| *Col3a1* | Mm01254476_m1 |
| *Acta2* | Mm00725412_s1 |
| *Vim* | Mm01333430_m1 |
| *Mmp9* | Mm00442991_m1 |
| *Timp1* | Mm00441818_m1 |
| *Mertk* | Mm00434920_m1 |
