# Supplementary material for: The Monocyte to Macrophage Transition in the Murine Sterile Wound
Source: PLoS One. 2014 Jan 22;9(1):e86660. doi: 10.1371/journal.pone.0086660 (PMC3899284; doi:10.1371/journal.pone.0086660)
Supplement: Table S2 — Number of wound monocyte/macrophage subsets in the wound. (DOCX) [file pone.0086660.s003.docx]

Table S2. Number of wound monocyte/macrophage subsets in the wound.

|  | Number of cells (10^4^ per animal) | | |
| --- | --- | --- | --- |
| Days after wounding | F4/80^+^ | Ly6C^hi^ | Ly6C^low^ |
| 1 | 14 ± 3 | 10.1 ± 2 | 1.4 ± 1 |
| 3 | 63.2 ± 25 | 24.2 ± 9 | 23.5 ± 9 |
| 7 | 95.2 ± 61 | 61.5 ± 40 | 15.3 ± 11.5 |
| 14 | 262 ± 70 | 137 ± 36 | 67.3 ± 25 |

Wound cell populations correspond to those shown in Figure 1A. Data are means ± SD, n = 3 mice per group, and are representative of at least 3 independent experiments.
