# Supplementary material for: The Monocyte to Macrophage Transition in the Murine Sterile Wound
Source: PLoS One. 2014 Jan 22;9(1):e86660. doi: 10.1371/journal.pone.0086660 (PMC3899284; doi:10.1371/journal.pone.0086660)
Supplement: Table S3 — Donor-derived cells recovered after sponge adoptive transfer. (DOCX) [file pone.0086660.s004.docx]

Table S3. Donor-derived cells recovered after sponge adoptive transfer.

|  | Number of CD45.1^+^ cells (10^3^ per animal) | |
| --- | --- | --- |
| CD45.1^+^ population | Post-transfer day 1 | Post-transfer day 7 |
| F4/80^+^ | 2.4 ± 0.9 | 2.9 ± 1.2 |
| F4/80^+^Ly6C^hi^ | 0.8 ± 0.4 | 0.06 ± 0.02 |
| F4/80^+^Ly6C^low^ | 0.7 ± 0.3 | 2.0 ± 0.9 |

Naive CD45.1 congenic mice received sponges for 1 day. Sponges from CD45.1 donor mice were then transferred to CD45.2 recipients and cells recovered from the wounds 1 to 7 days post-transfer. Donor-derived cells were identified by expression of CD45.1. Data shown are means ± SD, n = 3 mice per group.
